# A new species of *Tarsonops* (Araneae, Caponiidae) from southern Belize, with a key to the genera of the subfamily Nopinae

**DOI:** 10.3897/zookeys.289.4493

**Published:** 2013-04-12

**Authors:** Jason E. Bond, Steven J. Taylor

**Affiliations:** 1 Department of Biological Sciences and Auburn University Museum of Natural History, Auburn University, Auburn AL 36849; 2 Illinois Natural History Survey, University of Illinois at Urbana-Champaign, 1816 S. Oak Street (MC-652), Champaign IL 61820-6953

**Keywords:** Spider, *Tarsonops*, new species, Belize

## Abstract

A new species of Caponiidae, *Tarsonops irataylori* **sp. n.** is described from southern Belize, and a key to the genera of the subfamily Nopinae is provided.

## Introduction

Comprising 84 species parceled among 15 genera (Platnick 2012), the spider family Caponiidae is widely distributed but not currently known from Australia or Europe. Petrunkevitch (1939) divided the Caponiidae into two subfamilies, Caponiinae and Nopinae, the latter of which is characterized by subsegmented tarsi and has a New World distribution with 53 species described from North, South, and Central America and numerous species known from the Caribbean. While recent authors have questioned the monophyly of the Caponiinae, citing a lack of synapomorphies (Jiménez et al. 2011), the Nopinae is generally regarded as a valid group with subsegmented tarsi as a distinguishing feature. However, Platnick (1994) suggests that caponine taxa with fewer than eight eyes may be more closely related to nopines.

The genus *Tarsonops*, the subject of this paper, was erected by Chamberlin (1924) to accommodate the species, *Nops sternalis*, originally described by Banks (1898). At the time, Chamberlin (1924) also described, on the basis of female specimens, three additional species, *Tarsonops clavis*, *Tarsonops sectipes*, and *Tarsonops systematicus*, all collected in Mexico adjacent to the Gulf of California. He also provided a key to species based on female anatomy, with an emphasis on leg morphology. Subsequently, Gertsch (1935) published additional records for *Tarsonops systematicus*, collected in southern Texas, and Ubick (2005) reports that this species also occurs in California and Arizona and illustrates the male pedipalp (figure 18.10). Although numerous new species have been described in the family Caponiidae, including its subfamily Nopinae since the 1930s, no new species of *Tarsonops* have been described.

The primary purpose of this paper is to describe a new species of *Tarsonops* collected from Belize and to provide a key for the nopine genera. Unfortunately, this newly discovered species is known from only a single specimen. While a large of number of new species are described only from single specimens, greater than 1/6^th^ of all species (Lim et al. 2012), it is with some trepidation that we propose a new taxon on the basis of a single unique specimen. However, the morphological uniqueness of the species, the extension of the genus distribution, and recognition of important species level and morphological diversity serves as the impetus despite any misgivings. Moreover, it may very well be, given the combination/absence of characteristics (Table 1) for this species, that it may ultimately represent a new genus or species group, however, its palpal morphology closely resembles that described for *Tarsonops systematicus* by Ubick (2005). Although two of the nopine genera are monotypic (*Nopsides* Chamberlin, 1924 and *Nyetnops* Platnick & Lise, 2007) it is our opinion that the description of a new genus should be postponed until more material, including the female, and potential other species become available.

**Table 1.** Character states for genera of Nopinae (Caponiidae) compared to *Tarsonops irataylori* sp. n.<br/>

	**Taxon**						
**Character**	*Cubanops*	*Nops*	*Nopsides*	*Nyetnops*	*Orthonops*	*Tarsonops* (all other species)	*Tarsonops irataylori* sp. n.
Number of eyes	2	2	4	2	2	2	2
Ventral translucent keel on the anterior metatarsi and translucent extension of the membrane between the anterior metatarsi and tarsi	yes	yes	no	no	yes	yes	no
Distally expanded endites	no	no	yes	yes	no	no	no
Patterned carapace	yes	no	no	yes	no	no	no
Dorsally extended inferior claw	no	yes	yes	no	no	no	no
Wide labium	yes	no	no	no	no	no	no
Bisegmented metatarsi IV	yes	yes	?	no	no	no	no
Palpal bulb longer than cymbium, distinction evident between bulb and embolus only by differences in cuticular surface	no	no	no	yes	no	no	no
Anterior tarsus with a distinct suture dividing it into two, the distal of which is shorter (versus anterior tarsus with several false sutures, most distinct of which is proximal	yes	yes	yes	yes	yes	no	no

### Key to the genera of the subfamily Nopinae (Caponiidae)

**Table:** 

1	4 eyes	*Nopsides* Chamberlin, 1924
1’	2 eyes	2
2	Palpal endites (both sexes) expanded anteriorally, broadest at anterior apex of labium (see Platnick & Lise 2007); palpal bulb longer than cymbium, distinction between bulb and emobolus not evident except by sculpturing	*Nyetnops* Platnick & Lise, 2007
2’	Palpal endites (both sexes) not broadest anterior to apex of labium; normal palpal bulb with distinct embolus	3
3	Anterior tarsus with distinct suture that divides article into two distinct sub-segments	4
3’	Anterior tarsus with several false sutures, lacking distinct suture, not divided into two distinct sub-segments	*Tarsonops* Chamberlin, 1924
4	Metatarsus IV divided into two distinct subsegments	5
4’	Metatarsus IV entire	*Orthonops* Chamberlin, 1924
5	Tarsus I with inferior claw extended dorsally between superior claws; carapace generally lacking distinct patterning	*Nops* MacLeay, 1839
5’	Tarsus I with inferior claw not extending dorsally between superior claws; carapace patterned	*Cubanops* Sánchez-Ruiz et al., 2010

## Materials and methods

All measurements were taken with a Leica MZ16.5 stereomicroscope equipped with a 10× ocular and ocular micrometer scale. We measured the left appendage, usually in retrolateral view, using the highest magnification possible. Legs I-IV (femur, patella, tibia, metatarsus, tarsus) and palp article lengths (femur, patella, tibia, cymbium) given in order of proximal to distal. Illustrations were prepared using a Visionary Digital Imaging System (Ashland, VA). Photographs were recorded in multiple focal planes and assembled using the Zerene Stacker software package (Zerene Systems LLC, Richland, WA). The habitus illustration was constructed from whole body images that were bisected, copied, and reflected in Adobe Photoshop (Adobe Systems, Inc.) to produce a roughly symmetrical image (technique described in Bond 2012). Measurements in millimeters.

## Taxonomy

### Tarsonops irataylori sp. n.

urn:lsid:zoobank.org:act:4B93D052-EA8C-43E7-A5D4-28A52FE05DBE

http://species-id.net/wiki/Tarsonops_irataylori

Map 1 , Figs 1 - 6

#### Type material.

**Figures 1–3.**
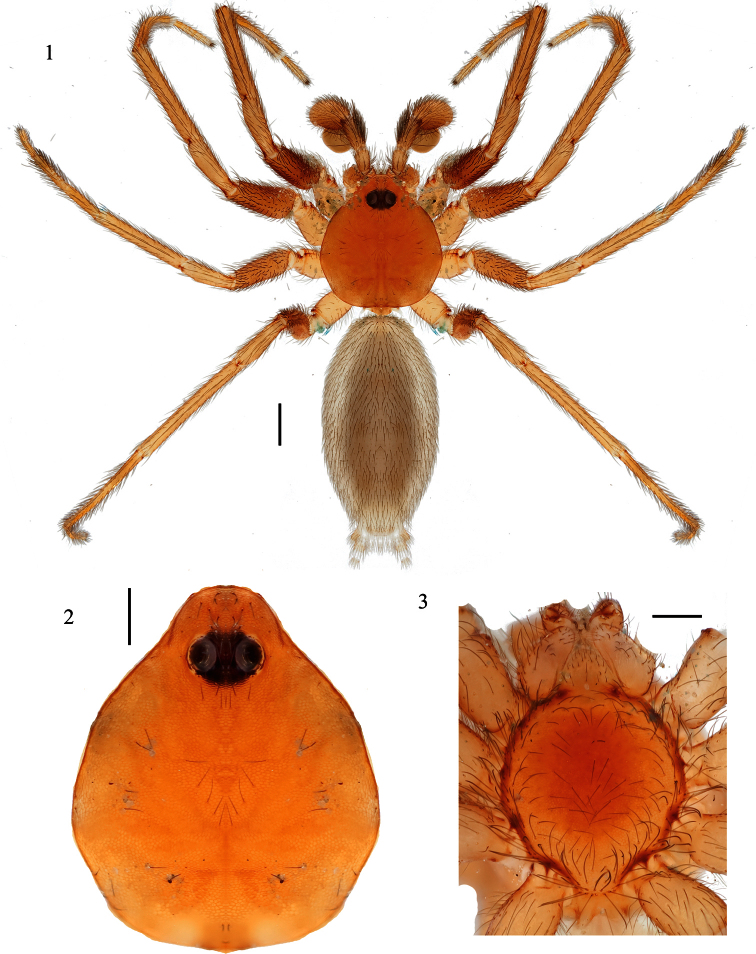
*Tarsonops irataylor* sp. n., male holotype **1** habitus, dorsal view **2** carapace, dorsal view **3** cephalothorax, ventral view. Scale bar = 0.50 mm (Fig. 1); 0.25 mm (Figs 2, 3)

**Holotype** male from BELIZE: **Toledo District**: Cave near Pueblo Creek Cave: 37 km WNW of Punta Gorda, 16°12'N, 89°08'W (Figure 1): 16 April 2011: sjt11-018: Coll. Michael E. Slay, Jean K. Krejca, Christy M. Slay, Geoffrey B. Hoese, Germano Coe. Sample# 253, Specimen# 0222. On dry flowstone in entrance zone, 0.1 lux, air temperature 25.7 °C, soil temperature 23.5 °C, relative humidity 91.2%. Deposited in the Auburn University Museum of Natural History collection.

**Map 1.**
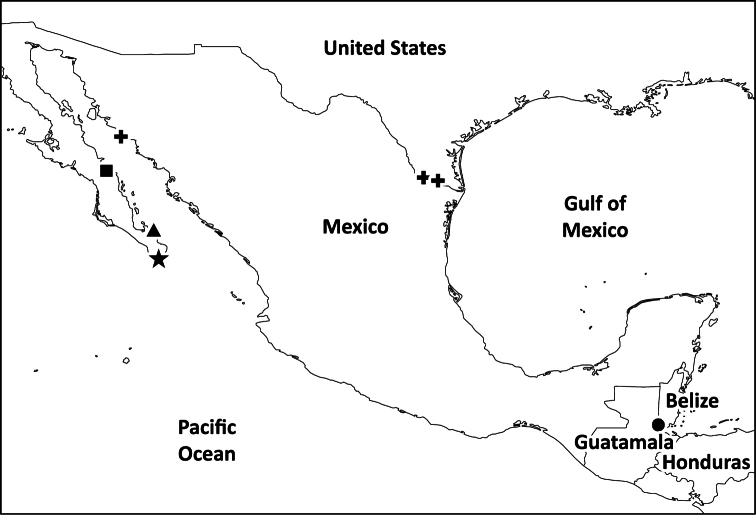
Distribution of *Tarsonops* species: *Tarsonops sternalis* (star), *Tarsonops sectipes* (triangle), *Tarsonops clavis* (square), *Tarsonops systematicus* (crosses, also recorded from California and Arizona, see Ubick et al. (2005)), *Tarsonops irataylori* sp. n. (circle).

#### Etymology.

The specific epithet honors the contributions of Mr. Ira W. Taylor to the study of subterranean ecosystems.

**Diagnosis.** *Tarsonops irataylori* sp. n. differs from all known species of *Tarsonops* by the absence of a ventral translucent keel on the anterior metatarsi and a highly reduced translucent extension of the membrane between the anterior metatarsi and tarsi.

#### Description of male holotype. 

**Figures 4–6.**
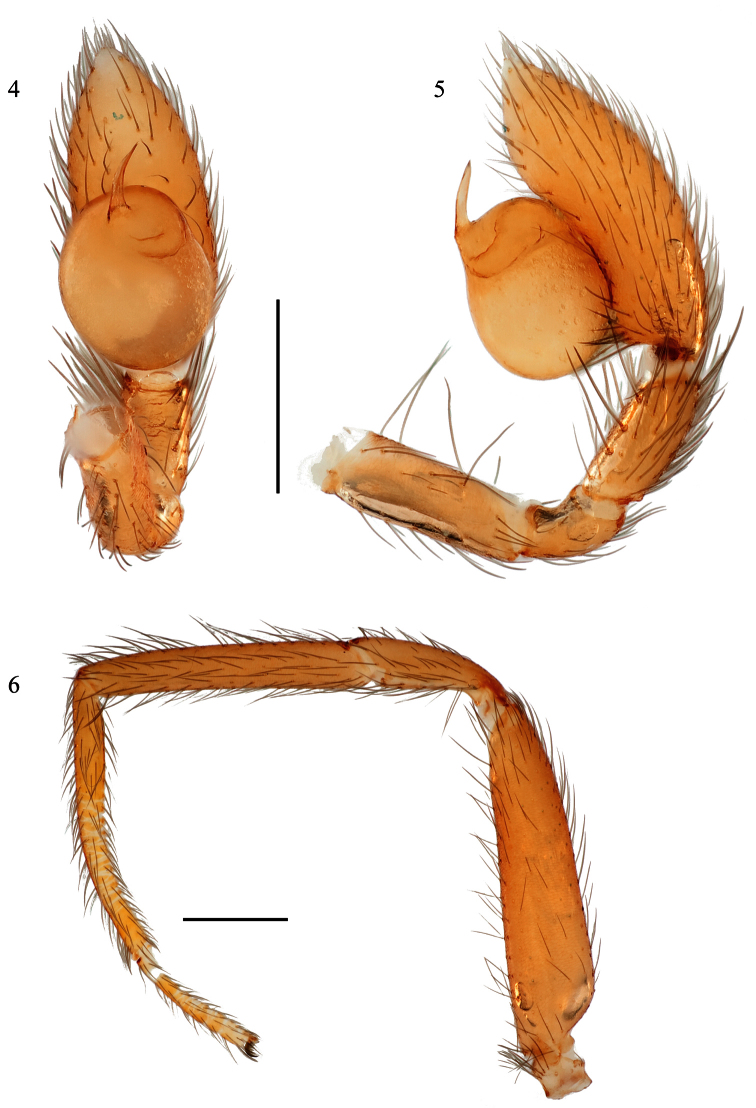
*Tarsonops irataylor* sp. n., male holotype. **4** left pedipalp, ventral view **5** left pedipalp, retrolateral view **6** leg I, retrolateral view. Scale bars = 0.50 mm.

*Specimen preparation and condition*. Specimen collected live, preserved in 70% ethanol. Coloration may be faded. Pedipalp, leg I left side removed and stored in vial with specimen. *General coloration*. Carapace, chelicerae, legs light orangish red (Figs 1, 2). Abdomen uniform very pale grayish brown dorsally. No dorsal carapace or abdominal patterning. *Cephalothorax*. Carapace 1.56 long, 1.40 wide, with sparse thin setae, surface lightly granular (Fig. 3), pars cephalica elevated slightly. Clypeus height 1.5× eye diameter. Two eyes, eyes separated by distance equal to radius. Sternum lightly setose, widest between coxae II, III (Fig. 4). Sternum length 1.12, width 1.00. Palpal endites rectangular, anterior margin rounded, extending slightly beyond anterior margin of labium (Fig. 4). Labium width 0.348, length 0.244. *Legs*. Leg I: 1.67, 0.740, 1.34, 1.41, 0.626; Leg II: 1.672, 0.751, 1.335, 1.485, 0.568; Leg III: 1.401, 0.600, 1.120, 1.404, 0.720; Leg IV: 1.814, 0.663, 1.509, 2.000, 1.028. Legs I-IV metatarsi and tarsi subsegmented distally (Fig. 6). Superior tarsal claw, Leg I with 5 teeth; inferior tarsal claw not extending dorsally between superior tarsal claws. Tarsus I with two trichobothria. Metatarsus I with 4 trichobothria, arranged along dorsal midline, lacking a ventral translucent keel, translucent extension of the membrane between the anterior metatarsus I and tarsus I greatly reduced, barely evident on close examination as wrinkled bump. Leg I illustrated in Figure 6. *Pedipalp*. (Figs 5, 6): 0.522, 0.270, 0.357, 0.940; bulb total length 0.618. Dense group of setae on prolateral tibial surface. Embolus short, less than 1/4^th^ length of bulb, tapering to sharp single point, bulb sub-spherical.

## Discussion

Although this species was taken from just inside a cave, it does not exhibit any obvious troglomorphies, and may be accidental in this habitat. The description of *Tarsonops irataylori* sp. n. extends the range of the genus 9 degrees east and 6.8 degrees south from the previously known range. A number of undescribed species of *Tarsonops* are known from collections in Mexico (Platnick, pers. comm. 31 October 2011).

*Tarsonops irataylori* sp. n. is the first species of *Tarsonops* described which lacks a ventral translucent keel on the anterior metatarsi and marked translucent extension of the membrane between the anterior metatarsi and tarsi. Chamberlin’s (1924) diagnoses of the genus does not list these characters, thus we have taken the conservative approach of placing the species in this genus. As discussed above, future studies in which more specimens are examined, may further warrant the establishment of a new genus to accommodate this somewhat unusual species.

## Supplementary Material

XML Treatment for Tarsonops irataylori

## References

[B1] Banks N (1898) Arachnida from Baja California and other parts of Mexico. Proceedings of the California Academy of Sciences (3) 1: 205–308.

[B2] Bond JE (2012) Phylogenetic treatment and taxonomic revision of the trapdoor spider genus *Aptostichus* Simon (Araneae, Mygalomorphae, Euctenizidae) Zookeys 252: 1–209. doi: 10.3897/zookeys.252.3588 PMC3560839 23378811

[B3] Chamberlin RV (1924) The spider fauna of the shores and islands of the Gulf of California. Proceedings of the California Academy of Sciences, Fourth Series 12 (28): 561-694.

[B4] Gertsch WJ (1935) Spiders from the southwestern United States. American Museum Novitates 792: 1-31.

[B5] Jiménez ML Platnick NI Dupérré N (2011) The Haplogyne spider genus *Nopsides* (Araneae, Caponiidae), with notes on *Amrishoonops*. American Museum Novitates 3708: 1-18. doi: 10.1206/3708.2

[B6] Lim GS Balke M Meier R (2012) Determining Species Boundaries in a World Full of Rarity: Singletons, Species Delimitation Methods. Systematic Biology 61: 165-169. doi: 10.1093/sysbio/syr030 21482553

[B7] Petrunkevitch A (1939) Classification of the Araneae with key to suborders and families. Transactions of the Connecticut Academy of Arts and Sciences 33: 139-190.

[B8] Platnick NI (1994) A review of the Chilean spiders of the family Caponiidae (Araneae, Haplogynae). American Museum Novitates 3113: 1-10.

[B9] Platnick NI (2012) The World Spider Catalog, ver. 12.5, The World Spider Catalog. URL http://research.amnh.org/iz/spiders/catalog/INTRO1.html

[B10] Platnick NI Lise AA (2007) On *Nyetnops*, a new genus of the spider subfamily Nopinae (Araneae, Caponiidae) from Brazil. American Museum Novitates 3595: 1-9. doi: 10.1206/0003-0082(2007)3595[1:ONANGO]2.0.CO;2

[B11] Ubick D (2005) Caponiidae. In: Ubick D, Paquin P, Cushing PE, Roth V (Eds) Spiders of North America: an identification manual. American Arachnological Society. 75–76.

